# Immunosenescence and cancer: Opportunities and challenges

**DOI:** 10.1097/MD.0000000000036045

**Published:** 2023-11-24

**Authors:** Zhibin Fu, Hailong Xu, Lanping Yue, Weiwei Zheng, Linkang Pan, Fangyi Gao, Xingshan Liu

**Affiliations:** a Weifang Hospital of Traditional Chinese Medicine, Shandong University of Traditional Chinese Medicine, Weifang, Shandong, China; b Weifang Hospital of Traditional Chinese Medicine, Weifang, Shandong, China.

**Keywords:** aging, immunosenescence, senescence, tumor microenvironment

## Abstract

As individuals age, cancer becomes increasingly common. This continually rising risk can be attributed to various interconnected factors that influence the body’s susceptibility to cancer. Among these factors, the accumulation of senescent cells in tissues and the subsequent decline in immune cell function and proliferative potential are collectively referred to as immunosenescence. Reduced T-cell production, changes in secretory phenotypes, increased glycolysis, and the generation of reactive oxygen species are characteristics of immunosenescence that contribute to cancer susceptibility. In the tumor microenvironment, senescent immune cells may promote the growth and spread of tumors through multiple pathways, thereby affecting the effectiveness of immunotherapy. In recent years, immunosenescence has gained increasing attention due to its critical role in tumor development. However, our understanding of how immunosenescence specifically impacts cancer immunotherapy remains limited, primarily due to the underrepresentation of elderly patients in clinical trials. Furthermore, there are several age-related intervention methods, including metformin and rapamycin, which involve genetic and pharmaceutical approaches. This article aims to elucidate the defining characteristics of immunosenescence and its impact on malignant tumors and immunotherapy. We particularly focus on the future directions of cancer treatment, exploring the complex interplay between immunosenescence, cancer, and potential interventions.

## 1. Introduction

With the prolongation of lifespan, people are facing higher risks of age-related diseases, especially cancer.^[1,2]^ The elderly population also faces higher rates of immune treatment failure and posttreatment relapse, giving rise to the concept of “immunosenescence”. Immunosenescence was initially described by Roy Walford and involves several key components: remodeling of the immune organs, age-related impaired innate and adaptive immune functions, impaired vaccine responses, and increased susceptibility to infections and malignancies.^[3–5]^

The immune system plays a critical yet paradoxical role in cancer, acting both as an important defender against tumors and sometimes promoting tumor development.^[6]^ This intricate relationship is further complicated by the natural aging process of the immune system. Immunosenescence is a natural process in which the immune function in the human body gradually declines with age and significantly impacts an individual’s susceptibility to diseases, including cancer. The main characteristics of immunosenescence include a decline in adaptive immunity, reduced resistance to infections, and an increased risk of autoimmune diseases.^[5,7]^ Restoring the aging immune system in elderly cancer patients through immune therapy holds promising prospects, but it faces a major obstacle known as the tumor microenvironment (TME). In the context of innate and adaptive immunity, numerous studies have revealed differences in tumor response between young and old individuals, but the underlying mechanisms still require further research.

Despite significant progress in understanding immunosenescence and searching for interventions related to age-related immune decline,^[8–10]^ there are still ongoing challenges and opportunities for continuous innovative research and interventions due to the complex network of interactions within the immune system and its relationship with the TME. In this article, we primarily outline the characteristics of immunosenescence, followed by a discussion of the interactions between immune cells and cancer cells within the aging TME. Finally, we provide an overview of future interventions based on immunosenescence.

## 2. Concept and markers of immunosenescence

Immunosenescence is a complex biological process involving multiple mechanisms at the organ and cellular levels.^[11]^ This process results in a decreased immune system function, leading to reduced responses to infections and vaccines in older individuals. While the complete scope of these biological changes remains not entirely understood, several distinct alterations have been observed: thymic atrophy, impaired stem cell function, disruption of the balance between naïve and memory cells in the T and B cell populations, chronic inflammation, accumulation of senescent cells, impaired neoantigen response, mitochondrial dysfunction, genomic instability, and stress response.^[8,12–14]^ Understanding the markers and features associated with immunosenescence is essential, particularly in relation to age-related diseases.

Thymic involution plays a crucial role in shaping the balance of immune cells, especially T cells.^[9,15,16]^ The thymus consists of 2 distinct tissue types: epithelial tissue, which has thymic developmental functions, and non-epithelial space surrounding blood vessels that lacks thymic developmental functions. With thymic involution, the epithelial space gradually decreases while the non-epithelial space becomes more prominent in aging thymus. This transition leads to fewer newly generated naïve T cells, more peripheral memory T cells, and reduced migration of newly generated T cells to peripheral tissues.^[17–19]^

Inflammation is an important characteristic of immunosenescence, indicating a state of persistent low-grade systemic inflammation, as evidenced by elevated inflammatory markers in the bloodstream.^[20]^ Cellular senescence plays a significant role in the process of inflammaging, and in the context of immunosenescence, senescent CD8 cells become a key driver of inflammaging within the body.^[21]^ Senescent cells display a distinctive senescence-associated secretory phenotype (SASP), contributing to the inflammaging phenotype by releasing various soluble factors, including interleukin-1 (IL-1), IL-6, IL-8, IL-13, IL-18, tumor necrosis factor, and their receptors.^[22–25]^ Understanding the intricate interactions between cellular senescence, pro-inflammatory factors, and aging is not only crucial for unraveling the mechanisms of immunosenescence but also for potentially devising interventions to mitigate the adverse effects of inflammaging and promote healthier aging.

## 3. Immunosenescence and cancer

The risk of malignant tumors increases with the individual’s age. While genetic mutations have long been considered an important factor in tumor development, recent research indicates that immunosenescence may also have a substantial impact in this process. It is worth noting that a study conducted by DeSantis et al^[26]^ revealed an increased risk of tumor incidence in the elderly population. Palmer et al^[27]^ analyzed 100 different types of tumors and proposed the view that the immune system has a significant impact on tumor development, further emphasizing the association between aging and cancer.

In the complex TME, many factors can trigger immune cell senescence, profoundly affecting their function. In elderly individuals, immune system dysregulation might encourage tumor growth and result in T-cell senescence, along with a notable rise in the number of tumor-associated macrophages and regulatory T cells (Tregs).^[28–32]^ Tumor cells trigger the PKA-CREB and P38 pathways through the production of endogenous cyclic adenosine monophosphate, leading to DNA damage and T-cell senescence.^[33–35]^ Activation of the ATM and AMPK pathways due to glucose exposure can also result in T-cell senescence.^[12,36]^ Furthermore, senescent T cells shift towards anaerobic glycolysis for energy generation, causing mitochondrial dysfunction and higher production of reactive oxygen species.^[37]^ Signal pathways such as NFκB, C/EBPβ, and cGAS-STING are also closely associated with T-cell senescence.^[38]^

Studies have demonstrated the critical role of CD8 T-cell immunosenescence in both tumor development and treatment.^[39]^ Age-related alterations in immune response have been observed in aged mice, including reduced expression of interferon (IFN) signaling in CD8 T-cells.^[40]^ The TME is significantly influenced by the aging process, with a demonstrated impact on the metabolism of T-cells within it, closely linked to tumor metastasis and infiltration. Age-related changes, such as matrix remodeling induced by hyaluronic acid and proteoglycan link protein 1, have been shown to enhance the invasiveness and distant metastasis propensity of melanoma cells.^[41]^ Elevated levels of age-related secreted frizzled-related protein 2 by fibroblasts promote angiogenesis and metastasis of melanoma.^[42]^ Elevated serum methylmalonic acid levels in elderly individuals stimulate SOX4 expression, leading to the transcriptional reprogramming of cancer cells and enhancing their invasiveness.^[43]^ Interestingly, some studies have found that aging may contribute to improved cancer prognosis, as evidenced by slower tumor proliferation in aged mice observed in various mouse models.^[44,45]^ Retrospective studies have highlighted that older patients with cancers such as colorectal cancer and lung cancer may experience slower tumor growth and reduced metastasis, possibly due to age-related factors impeding aggressive tumor growth.^[46,47]^ In certain cancer mouse models, such as B16F10 melanoma, the aged group exhibits smaller tumor volumes and higher survival rates.^[44]^ These diverse research findings collectively emphasize the intricate relationship between aging, immunosenescence, and tumor development, revealing the multifaceted impacts of age on cancer progression and prognosis.

## 4. Senescence-related immune cell subsets in the TME

### 4.1. Cancer-associated fibroblasts

Detecting cancer-associated fibroblasts (CAFs) poses a formidable hurdle owing to their inherent diversity and the absence of distinct fibroblast-specific indicators.^[48]^The accumulation of CAFs in the TME is associated with poor prognosis, primarily because CAFs produce immunosuppressive cytokines, including IL-6, CXC chemokine ligands, and TGFβ.^[49]^ CAFs mainly originate from bone marrow progenitor cells and surrounding adipose tissue.^[50,51]^ Various components of the SASP, such as TGFβ family ligands, IL-6, IL-1, and CXC chemokine ligands-1, may contribute to the formation and activation of CAFs.^[52–54]^ The accumulation of senescent cells and the secretion of SASP components may enhance the recruitment or formation of CAFs, ultimately creating an immunosuppressive environment.^[54]^ This immunosuppressive environment may weaken the effectiveness of immune checkpoint inhibitors (ICIs) as it harbors factors that impede immune responses. Additional investigation is required to unravel the impact of aging on the phenotype of CAFs, which is a challenging field. When stimulated by pro-inflammatory cytokines such as IL-1α, IL-1β, and tumor necrosis factor α, CAFs tend to shift towards a senescent state, inhibiting tumor cell apoptosis and promoting tumor cell dissemination. By releasing IL-6 and IL-8, and amphiregulin, senescent fibroblasts have been shown to affect the tumor microenvironment by promoting monocyte differentiation into pro-inflammatory M2 macrophages and driving PD-L1 expression in tumor cells.^[55,56]^ The impact of senescent fibroblasts of human aging on the tumor immune response through their SASP remains unclear and requires further investigation. Manipulating CAFs and cellular aging offers a promising strategy for alleviating tumor growth, but a deeper understanding of aging and how it influences the accumulation of CAFs and shapes their secretory profile in the complex tumor microenvironment is necessary.

### 4.2. T cells

In a person’s lifetime, although the total number of T-cells remains relatively stable, these subgroups undergo significant changes: the number of naive T-cells decreases, while highly differentiated CD28 memory T-cells or senescent cells increase.^[57]^ CD4 T-cells exhibit a stable diversity and production. On the contrary, CD8 T cells undergo notable age-related transformations,^[58]^ including marked alterations in surface molecule expression, such as a significant decrease in the CD28 co-stimulatory factor.^[59]^ Additionally, the elevated expression of immune markers such as CD57, Tim-3,and KLRG-1 is correlated with T-cell senescence.^[60–62]^ The heightened expression of inhibitory receptors on senescent T cells shares similarities with T cell exhaustion, sharing some functional and phenotypic characteristics,^[63,64]^ while these 2 processes possess separate regulatory mechanisms and distinct developmental attributes. It is worth emphasizing that the current understanding of T-cell senescence mainly comes from studies on peripheral blood, representing only a small fraction (2%) of the total T cell population.^[65]^ To delve further into the comprehension of T-cell senescence and immune senescence, further research is necessary that goes beyond peripheral blood analysis, taking into account the complex dynamics and heterogeneity that exist within different T cell subgroups.

Naive T cells have significant implications in the field of immunosenescence research because they rely entirely on thymic function for their generation. Thymic degeneration results in a substantial reduction in the production of thymic naive T cells, thereby decreasing the diversity of T cell antigen receptors.^[15,66]^ This reduction in diversity ultimately disrupts T cell homeostasis and serves as the basis for aging-related adaptive immune system failure.^[67]^ Understanding the intricate relationship between thymic degeneration and immunosenescence is crucial for elucidating the mechanisms behind the observed decline in adaptive immune responses with increasing age.

Memory T cells gradually accumulate during the senescent process.^[68,69]^ Among them, memory T cells with a naive phenotype also accumulate with increasing age.^[70]^ In the aging population, there is often an increased quantity and ratio of memory CD8 T cells, which can have a substantial impact on immune function.

Tregs are a subset of immunosuppressive CD4 T cells, commonly distinguished by the presence of Foxp3 and CD25 expression.^[71]^ In aging mice, Tregs often experience an increase in their numbers and demonstrate heightened suppressive capabilities, more effectively limiting the growth of effector T cells compared to Tregs in young mice.^[72]^ Tregs in aging mice show overexpression of IL-10, effectively inhibiting the expression of antigen-presenting receptor CD86 in dendritic cells.^[73]^ However, Tregs expressing cell senescence markers p16 and p21 in aging mice exhibit lower efficiency in suppressing T cell activation in comparison to the youthful control group.^[74]^ Researchers are still debating the effects of cellular senescence on Tregs, and further investigation is needed to unravel the complexity of these interactions.

### 4.3. Myeloid-derived suppressor cells (MDSCs)

There has been a noted rise in MDSCs within the blood and bone marrow of elderly mice bearing tumors, and this accumulation could potentially hinder the clearance of tumor cells.^[75]^ Senescent cells with a SASP release various chemokines and cytokines, promoting immune evasion and tumor cell metastasis.^[75]^ The expansion of MDSCs may not only promote immunosenescence but also induce harmful aging-related effects in host tissues by secreting TGF-β and IL-10.^[76]^ In aged mice, increased tumor growth is associated with higher levels of MDSCs and Arginase1 in tumor tissues.^[77]^ While the total proportion of MDSCs in the bone marrow and spleen of aged animals increases, their functionality remains intact.^[78]^ Treg depletion efficiently delays tumor growth in young mice, but this effect is not observed in aged mice, mainly due to increased recruitment of MDSCs.^[79]^ Elevated levels of circulating MDSCs have been documented in melanoma patients and are linked to resistance against anti-CTLA-4 therapy.^[80]^ These findings suggest that phenotypic changes and increased MDSC numbers in the elderly may impair the efficacy of immunotherapy.

### 4.4. Tumor-associated macrophages (TAMs)

Macrophages express markers of senescence during the aging process, including p16 expression and SA-β-gal positivity.^[81]^ In the TME, TAMs play a driving role in angiogenesis, tumor growth, and metastasis through the production of cytokines like IL-6 and IL-10. This behavior is similar to the SASP.^[82]^ Studies have shown that aged TAMs accelerate the growth of mesothelioma in elderly mice,^[83]^ while inhibiting these TAMs with F4/80 antibodies can reinstate the effectiveness of immunotherapy in elderly mice.

### 4.5. Other cells

Dendritic cells exhibit decreased functionality in older individuals, including antigen presentation, endocytic activity, and interferon production.^[84]^ The phagocytic capability of neutrophils weakens with increasing age.^[85]^ B cells are crucial for antibody production and immune response, and immunosenescence can affect the components of B cells.^[86,87]^ In mice, the replenishment of mature splenic B cells diminishes as they age, which can potentially impact antibody production and overall immune function.^[88]^

## 5. Immunosenescence and tumor immunotherapy

As the elderly population continues to expand, the significance of immunotherapy in cancer treatment for older patients is on the rise. However, there are variations in how effective immunotherapy is in older versus younger patients, influenced by factors such as cancer type, disease stage, and comorbidities. The connection between age and immune-related side effects is still a subject of debate. Examining the effectiveness of immunotherapy in older cancer patients is vital for improving treatment approaches and addressing the specific challenges presented by aging and cancer. Here, we delve into the complex landscape of cancer immunotherapy, with a focus on its efficacy in the elderly population and the corresponding considerations.

Immunosenescence can impact the effectiveness of ICIs treatment in cancer patients, as observed in cases of non-small cell lung cancer.^[89]^ Preclinical studies have revealed that the response to immune checkpoint blockade therapy is less effective in aged mouse models compared to young control groups, highlighting the important impact of age on treatment outcomes.^[90]^ Immune checkpoint blockade antibody therapy has become a treatment method for various cancer types, therefore it is necessary to understand the impact of age on the efficacy of different tumor malignancies. Currently, research and clinical trials examining how a patient’s age relates to the outcomes of ICIs treatment are still somewhat limited. Furthermore, the impact of the specific tumor microenvironment in older patients on their response to immune therapy remains a subject that requires further exploration.

A study involving aged mice with triple-negative breast cancer indicated limitations in the efficacy of ICIs. In a mouse model of melanoma, the efficacy of anti-CTLA-4, anti-PD-1, and anti-PD-L1 antibodies varied with age, with a more favorable therapeutic effect observed in the elderly group.^[91]^ Meta-analyses of randomized trials have found that ICIs demonstrate efficacy in both young and elderly patients, and the survival status for older individuals may even be better than that of the control group intervention,^[92–95]^ although some older patients may experience fatal toxic effects.^[96]^ Another study indicated that ICIs could potentially be more effective and well-tolerated in older patients with advanced melanoma.^[97]^Changes in the TME, including a decrease in interferon signaling and antigen presentation, have been observed in elderly triple-negative breast cancer patients.^[40]^ Exploring the complex relationship between age and ICIs treatment outcomes, understanding age-specific changes in treatment response, and analyzing subtle differences in the tumor microenvironment are crucial steps in achieving tailored cancer immunotherapy for both young and elderly patient populations.

In most studies, the participation of elderly patients in clinical trials of ICIs is limited, resulting in insufficient data on treatment safety and toxicity for this age group. A comprehensive understanding of how ICIs treatment affects elderly patients remains elusive. To provide more personalized and effective cancer immunotherapy for the elderly, additional research and clinical trials are necessary to assess the impact, mechanisms, and safety of ICIs treatment in this age group.

## 6. Potential therapeutic strategies to improve responses among older adults

T-cell senescence has been identified as an important factor related to the aging process and immunesenescence, impacting the effective functioning of the immune system. senescent T cells, along with the overall immunosenescence environment, can collectively reduce the efficacy of adoptive cell transfer therapy. Simple antiaging strategies may not achieve the desired therapeutic outcomes and may potentially cause harm to healthy tissues, especially in elderly individuals. Recent research has questioned the idea of irreversible cell aging,^[98]^ as inhibiting the human telomerase reverse transcriptase gene can allow senescent cells to regain the capacity to reenter the cell cycle. Senescent cells that acquire stem cell-like traits display increased capacity for proliferation and potential to promote tumor growth. The impact of senescent T cells on immunosenescence, tumor growth, metastasis, and resistance to immunotherapy remains a subject of current research.

Recent studies have revealed the potential of drug interventions in slowing down the aging phenotype. Metformin, a widely used antidiabetic medication, research indicates that it has the potential to slow down the aging process.^[99]^ Positive impacts on aging have also been noted in mouse models, including C57BL/6 mice with relatively longer lifespan and genetically hybrid mice.^[100,101]^ Retrospective analysis of diabetic patients receiving metformin treatment has shown increased lifespan compared to individuals without diabetes.^[102]^

mTOR, is a protein kinase enzyme that plays a crucial regulatory role within cells, including cell growth and proliferation, cellular metabolism, autophagy regulation, immune system and longevity and lifespan.^[103]^ Gene regulation of mTOR signaling can slow down the aging process in various organisms.^[104–106]^ Rapamycin, when bound to FKBP12, disrupts mTORC1 and leads to its inhibition. This effective anticancer drug has shown prospects in slowing down or reversing various age-related changes.^[107–109]^ Rapamycin can reverse the enhanced SASP observed in senescent cells.^[110–112]^ Research on the mTORC1 pathway may be a feasible strategy against the aging process. However, rapamycin lacks selectivity and may result in significant side effects, including inflammatory reactions and harm to normal tissues.^[113]^ Therefore, concerns exist regarding its suitability as the optimal treatment method.

Immunotherapy, has delivered substantial clinical benefits to patients facing advanced or traditionally resistant cancers.^[114,115]^ The use of ICIs, which target specific proteins produced by senescent cells or block inhibitory receptor activation, remains controversial, primarily due to limited participation of elderly patients in clinical trials.^[116]^ Researchers suggest investigating chimeric antigen receptor T-cell cells that target age-specific surface antigens as a potential treatment approach. We have collected recent clinical immunotherapy trials targeting senescence-related biomarkers (Table 1).Recent clinical immunotherapy trials involving age-related biomarkers (including CD57 and KLRG-1) have not been extensively investigated. Recent research has identified the urokinase-type plasminogen activator receptor as a novel cell surface protein induced during the aging process. Urokinase-type plasminogen activator receptor-specific chimeric antigen receptor T-cell have demonstrated significant efficacy in eliminating senescent cells both in vitro and in vivo,^[117]^ but further research is needed to determine their safety in potential clinical applications.

**Table 1:** Current immunotherapy targeting senescence-associated biomarkers.

ClinicalTrials.gov identifier	Conditions	Interventions	Target	Trial phase	Status
NCT01426828	Multiple myeloma	CD3/CD28	KLRG-1	Phase 2	Completed
NCT04354246	Advanced cancer	COM902	TIGIT	Phase 1	Recruiting
NCT04150965	Relapsed refractory multiple myeloma	Pomalidomide, dexamethasone	TIGIT, LAG-3	Phase 2	Active
NCT04693234	Cervical cancer	Ociperlimab	TIGIT, PD-1	Phase 2	Active
NCT04995523	Non-small-cell lung carcinoma	AZD2936	TIGIT, PD-1	Phase 2	Recruiting
NCT04732494	Esophageal squamous cell carcinoma	Tislelizumab, Ociperlimab	TIGIT, PD-1	Phase 2	Recruiting
NCT03489343	Advanced solid tumor malignancies	Sym023	Tim-3	Phase 1	Completed
NCT05357651	Solid tumor, lymphoma	LB1410	Tim-3, PD-1	Phase 1	Recruiting
NCT04139902	Melanoma	TSR-022, TSR-042	Tim-3, PD-1	Phase 2	Recruiting
NCT03680508	Liver cancer	TSR-022, TSR-042	Tim-3, PD-1	Phase 2	Recruiting

Recent studies have deepened our understanding of the molecular mechanisms underlying the aging process, opening up new avenues for potential interventions. Surprisingly, research has revealed numerous genes that can extend lifespan, highlighting the remarkable plasticity inherent in the aging process. Genes governing aging exhibit a high degree of conservation in humans, suggesting that these pathways remain quite similar across considerable evolutionary distances. These findings offer exciting prospects for developing interventions in the aging process.

## 7. Conclusion and further perspectives

The increasing attention to aging is due to its significant impact on various age-related pathologies, particularly cancer. Despite growing evidence of its importance, the age of patients is often insufficiently considered in preclinical studies. Aging and cellular senescence lead to substantial alterations in the TME, resulting in the accumulation of various immunosuppressive cells. The presence of these immunosuppressive cells in the TME contributes to increased tumor cell resistance and greater potential for immune evasion. Understanding the intricate interplay between aging, cellular senescence, and the TME is crucial for developing more effective cancer treatment strategies and addressing the unique challenges posed by age-related factors.

Significant progress has been made in understanding age-related immune changes through immunosenescence research in mouse models. To gain a comprehensive understanding of immunosenescence, it is crucial to leverage existing immunological techniques and experimental advances to comprehensively explore the intricacies of the human immune system. In the face of a growing public health challenge posed by malignant tumors, gaining comprehensive insight into immunosenescence, its underlying mechanisms, and potential therapeutic targets has become paramount. Targeting immune senescence cells is considered a novel intervention strategy in cancer patients, holding promise for breakthroughs in cancer treatment.

## Author contributions

**Writing – original draft:** Zhibin Fu, Hailong Xu, Lanping Yue, Weiwei Zheng.

**Writing – review & editing:** Linkang Pan, Fangyi Gao, Xingshan Liu.
